# Modulation of New Excitons in Transition Metal Dichalcogenide‐Perovskite Oxide System

**DOI:** 10.1002/advs.201900446

**Published:** 2019-04-29

**Authors:** Xinmao Yin, Ming Yang, Chi Sin Tang, Qixing Wang, Lei Xu, Jing Wu, Paolo Emilio Trevisanutto, Shengwei Zeng, Xin Yu Chin, Teguh Citra Asmara, Yuan Ping Feng, Ariando Ariando, Manish Chhowalla, Shi Jie Wang, Wenjing Zhang, Andrivo Rusydi, Andrew T. S. Wee

**Affiliations:** ^1^ International Collaborative Laboratory of 2D Materials for Optoelectronics Science and Technology Shenzhen University Shenzhen 518060 China; ^2^ Department of Physics Faculty of Science National University of Singapore Singapore 117542 Singapore; ^3^ Singapore Synchrotron Light Source (SSLS) National University of Singapore Singapore 117603 Singapore; ^4^ Institute of Materials Research and Engineering A∗STAR (Agency for Science, Technology and Research) 2 Fusionopolis Way Singapore 138634 Singapore; ^5^ NUS Graduate School for Integrative Sciences and Engineering National University of Singapore Singapore 117456 Singapore; ^6^ Centre for Advanced 2D Materials and Graphene Research Centre National University of Singapore Singapore 117551 Singapore; ^7^ NUSNNI‐NanoCore National University of Singapore Singapore 117576 Singapore; ^8^ Energy Research Institute @ NTU (ERI@N) Research Techno Plaza X‐Frontier Block, Level 5, 50 Nanyang Drive Singapore 637553 Singapore; ^9^ Department of Materials Science and Metallurgy University of Cambridge Cambridge CB30FS UK

**Keywords:** 2D transition metal dichalcogenides, electronic correlations, excitons, heterointerfaces, perovskite oxides

## Abstract

The exciton, a quasi‐particle that creates a bound state of an electron and a hole, is typically found in semiconductors. It has attracted major attention in the context of both fundamental science and practical applications. Transition metal dichalcogenides (TMDs) are a new class of 2D materials that include direct band‐gap semiconductors with strong spin–orbit coupling and many‐body interactions. Manipulating new excitons in semiconducting TMDs could generate a novel means of application in nanodevices. Here, the observation of high‐energy excitonic peaks in the monolayer‐MoS_2_ on a SrTiO_3_ heterointerface generated by a new complex mechanism is reported, based on a comprehensive study that comprises temperature‐dependent optical spectroscopies and first‐principles calculations. The appearance of these excitons is attributed to the change in many‐body interactions that occurs alongside the interfacial orbital hybridization and spin–orbit coupling brought about by the excitonic effect propagated from the substrate. This has further led to the formation of a Fermi‐surface feature at the interface. The results provide an atomic‐scale understanding of the heterointerface between monolayer‐TMDs and perovskite oxide and highlight the importance of spin–orbit–charge–lattice coupling on the intrinsic properties of atomic‐layer heterostructures, which open up a way to manipulate the excitonic effects in monolayer TMDs via an interfacial system.

## 1 Introduction

Excitons are an intriguing class of electrically neutral electron–hole quasi‐particles, which can be formed by charge association or direct photoexcitation.^1^ The understanding of their underlying physical mechanisms in semiconductors has enabled for wide‐ranging applications such as organic light‐emitting diode technologies,^2^ laser media,^3^ and photovoltaic and solar cell systems.^4^ Furthermore, excitonic effects can potentially be utilized in optoelectronic devices in the ultraviolet to deep‐ultraviolet regime.^5^, ^6^

Many studies have reported novel physical phenomena in low‐dimensional transition metal dichalcogenides (TMDs) such as monolayer‐MoS_2_,^7^ with potential applications in electronic and optoelectronic devices,^8^ spintronics, and valleytronics.^9^ The strong many‐body interactions, highly prominent in 2D‐TMDs, are manifested in the form of excitonic complexes or resonant excitons.^10^ In comparison with excitons in bulk systems, reducing the system to a lower dimension strongly influences the nature and dynamics of electronic excitations.^11^ It is highly intriguing to generate new excitons in 2D‐TMD systems and thereby create novel applications based on this class of materials. This could possibly be realized by the manipulation of excitons from different materials across the interface. With a sandwich lattice structure that comprises three planes of 2D packed atoms that are covalently bonded in an X‐M‐X configuration (X: chalcogen; M: metal), it is therefore important to understand how excitons behave across 2D‐TMD interfaces.

Previous study has shown that high‐energy resonant excitons are present in the SrTi_1‐_ *_x_*Nb*_x_*O_3_ family.^12^ Intriguingly, the resonant excitons interact strongly with a graphene layer or manganite film via their interfacial interactions and thereby affect the electronic and optical properties of these materials.^13^, ^14^ It shows that the excitons from SrTiO_3_ (STO) substrate can even propagate ≈87 nm into the top manganite thin‐film systems.^14^ This provides an exciting opportunity to introduce and manipulate the propagation of excitons from 3D substrate materials to 2D‐TMDs. Given the unique properties of each class of materials, a heterostructure system that involves the stacking of 2D‐TMDs on a bulk perovskite oxide STO is expected to uncover unique many‐body behavior of new electronic excitations.^15^ However, in the case of 2D‐TMD heterostructure systems where many‐body effects play a pivotal role in the development of exotic physical phenomena, investigation into how excitonic activities could propagate via the heterointerface and the possible manipulation of this effect remains overlooked.

**Figure 1:**
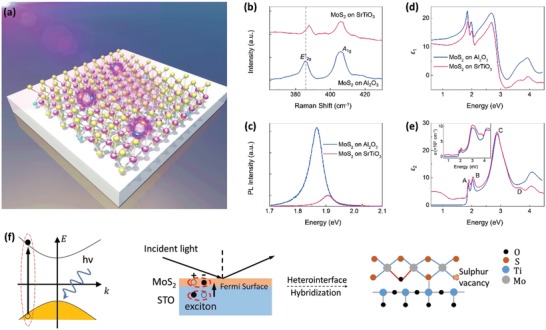
Optical characterization of monolayer‐MoS_2_ on Al_2_O_3_ and SrTiO_3_ substrate. a) High‐energy excitons (electron–hole pairs: blue–red pairs) for complex interplay between monolayer‐MoS_2_ on STO. Some sulfur atoms (yellow) are replaced by oxygen atoms (light blue). b) Raman spectra for MoS_2_/STO and MoS_2_/Al_2_O_3_. c) Photoluminescence spectra for MoS_2_/STO and MoS_2_/Al_2_O_3_. d,e) Dielectric functions d) ε_1_ and e) ε_2_ for MoS_2_/STO and MoS_2_/Al_2_O_3_. Inset: Absorption coefficient, α, spectra. f) Schematic of how electron–hole interactions are induced in monolayer‐MoS_2_ due to excitonic effects of the STO substrate.

In this study, we report the observation of new high‐energy excitons generated by a complex and unprecedented interplay of charge‐transfer mechanism of the monolayer‐MoS_2_ on STO heterointerface (**Figure** **1**a) via a detailed experimental and first‐principles study. Interestingly, a Fermi‐surface feature is observed at the MoS_2_/STO interface. Supported by first‐principles calculations, the mechanism leading to the anomalous electronic and optical properties of monolayer‐MoS_2_ is ascribed to the change in strong electronic correlations in monolayer‐MoS_2_ by the propagation of excitonic effect from the STO substrate, and couples with the interfacial orbital hybridization and spin–orbit coupling, which is different from the conventional mechanism of exciton related to the interband transitions. The observation of the Fermi‐surface feature in monolayer‐MoS_2_/STO has further implications on the appreciable effects that the excitonic effect propagation from the STO substrate plays a role in affecting the weak electronic correlations of the monolayer‐MoS_2_. The increased weak electronic correlations in MoS_2_ affect the Fermi surface instability that manifests itself in the form of the Fermi‐surface feature. Besides, the unique behaviors of these excitons such as their appearance in the UV energy region, anomalous scale of their binding energies, and their split into two excitons open new possibilities in device applications such as UV–vis range optoelectronics, light‐emitting devices, photovoltaics, and detectors.^5^, ^6^, ^16^ This study also shows the crucial role that many‐body interaction plays, which eventually leads to the control of excitonic effects in 2D‐TMDs via an interfacial system.

## 2 Results and Discussion

### 2.1 Observation of High‐Energy Exciton Peaks at the MoS_2_/STO Heterointerface

Large‐area monolayer‐MoS_2_ was synthesized on sapphire (Al_2_O_3_) substrate by the chemical vapor deposition (CVD) method using MoO_3_ and S powders as the reactants^17^ and then transferred onto other substrates using wet‐chemical method (details are provided in the Experimental Section). Figure 1b,c compares the Raman and photoluminescence (PL) spectra of monolayer‐MoS_2_ on Al_2_O_3_ (MoS_2_/Al_2_O_3_) with that of MoS_2_ transferred onto STO. The Raman spectrum of MoS_2_/Al_2_O_3_ in Figure 1b shows two main modes: $E_{2g}^{1}$ ‐mode (≈386 cm^−1^) due to opposing vibrations of in‐plane Mo and S‐atoms, and *A*_1g_‐mode (≈406.1 cm^−1^) due to opposing vibrations of two out‐of‐plane S‐atoms. The wavenumber difference between these two modes is ≈20.1 cm^−1^, consistent with previously reported for monolayer‐MoS_2_/Al_2_O_3_.^18^ This indicates that the CVD‐synthesized monolayer‐MoS_2_ is of high quality. Upon transferring monolayer‐MoS_2_ onto STO substrate, $E_{2g}^{1}$ ‐mode is blueshifted while *A*_1g_‐mode remains unchanged—indicating an increase in compressive strain between monolayer‐MoS_2_ and STO,^19^ which is attributed to the formation of shorter Mo—O bonds in monolayer‐MoS_2_ as demonstrated later. The compressive strain is also demonstrated in PL spectra in Figure 1c by the blueshift (≈40 meV) of the excitonic peak.^20^ It is observed that the Raman and PL intensities of MoS_2_/STO are significantly lower than the respective spectra of MoS_2_/Al_2_O_3_. This contrast in intensity highlights the strong influence optical interference and absorption effects have on the Raman and PL intensities of monolayer‐MoS_2_ on different substrates. Further observation of interfacial effects such as charge transfer at the MoS_2_/STO interface will be presented and discussed later.

Figure 1d,e displays the room‐temperature dielectric functions (ε(ω) = ε_1_(ω)+*iε*_2_(ω)) of MoS_2_/STO and MoS_2_/Al_2_O_3_ (inset: absorption coefficient) as measured by spectroscopic ellipsometry. This experimental technique utilizes the analysis of multilayer models^21^ such that the optical data of the top film layer are derived by eliminating the optical signals from the bottom layers or substrate. Hence, the final optical signal from the top sample layer is not a mere overlap of optical signals of both film and substrate. Instead, it registers the optical signal of the top film as a result of the interfacial influence from the bottom substrate (details are provided in the Experimental Section). In our analysis, the interface component contributes to the optical spectra of monolayer‐MoS_2_ (see the Experimental Section). Significant optical renormalization is observed in Figure 1e at the low (<1.7 eV), intermediate (1.7–3.3 eV), and high‐energy range (>3.3 eV). At low‐energy range, there is a nonzero offset in the ε_2_ and absorption spectrum of MoS_2_/STO while that of MoS_2_/Al_2_O_3_ falls to zero. This indicates the presence of carriers^22^ in MoS_2_/STO but absent in MoS_2_/Al_2_O_3_. The estimated effective number of carriers in monolayer‐MoS_2_ and interface is calculated from the optical conductivity spectra at energy range up to 1.5 eV,^22^, ^23^ standing at about 0.01 per MoS_2_ unit formula (details are provided in the Experimental Section). These carriers can be attributed to complex lattice–charge–orbit coupling as discussed later. At intermediate‐energy range, three main peaks labeled A (≈1.88 eV), B (≈2.04 eV), and C (≈3 eV) are observed for MoS_2_/Al_2_O_3_. They are related to the excitonic transitions^7^ at the K/K′ points (A and B) and the optical response due to band nesting^24^ between K and Γ points (broader peak C) in the Brillouin zone. A blueshift (≈40 meV) of peaks A and B is observed when monolayer‐MoS_2_ is transferred onto STO, consistent with the ≈40 meV blueshift of excitonic peak in PL spectra in Figure 1c. At both low and intermediate‐energy range, the spectral weight of MoS_2_/STO is always higher than that of MoS_2_/Al_2_O_3_. This spectral weight is transferred from the higher energy range (>3.3 eV). According to Zaanen–Sawatzky–Allen theory,^15^ such broad energy range spectral weight transfers occur due to strong local charge interactions and orbital hybridization in correlated systems. Thus, it indicates strong electron–hole interactions in MoS_2_/STO, significantly different from MoS_2_/Al_2_O_3_.

Interestingly, at high‐energy range, a new peak D appears at ≈3.8 eV for MoS_2_/STO. This peak is absent from monolayer‐MoS_2_ on Al_2_O_3_ and other substrates.^25^ As discussed later, this peak is attributed to a new resonant exciton induced by quantum propagation, in which the quantum wave function of the resonant exciton from the STO substrate^12^ propagates through the MoS_2_/STO interfacial layer by means of interfacial orbital hybridization that couples with the strong electron–hole interaction (Figure 1f). To further verify that peak D is not merely a result of overlapping optical signals of the film and substrate, an additional analysis that uses a referenced monolayer‐MoS_2_ to extract the changed STO dielectric functions is conducted. Analytical results clearly show that that this additional optical feature indeed arises as a result of the interfacial interactions between the MoS_2_ monolayer and STO substrate (details are provided in the Supporting Information).

### 2.2 First‐Principles Calculations to Confirm the High‐Energy Exciton Peaks

**Figure 2:**
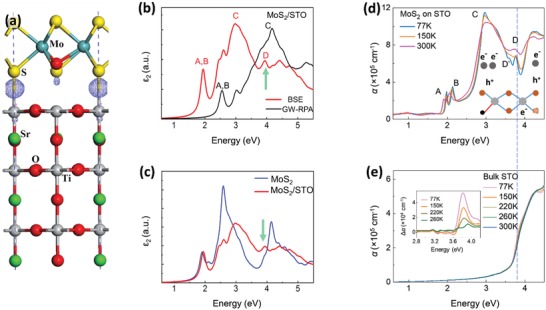
Optimized atomic structure, calculated optical spectra, and temperature‐dependent absorption coefficient spectra of MoS_2_/STO and bulk STO with DOS calculations of respective component orbital. a) The optimized atomic structure of MoS_2_ with a substitution O‐atom on the TiO_2_‐terminated STO [001] substrate with the differential charge density superimposed, in which the blue dots denote excess charge density and the pink dots denote depleted charge density. The differential charge density is visualized by an isosurface value of 1.0 × 10^−3^ e Å^−3^. b) The optical spectra of the MoS_2_ monolayer on the STO substrate calculated using GW‐RPA (without e–h interaction) and GW‐BSE (with e–h interaction) methods. c) GW‐BSE calculated optical spectra of the MoS_2_ monolayer with and without the STO substrate. d,e) α(ω) from 0.4 to 4.5 eV as a function of temperature of d) MoS_2_/STO and e) STO bulk with a dashed line serving as visual guide. (Inset: Differential absorption coefficient, *Δα*(ω,*T*) = α(ω,*T*)−α(ω,300 K).)

First‐principles calculations are performed to further understand the interfacial interaction between monolayer‐MoS_2_ and STO (details are provided in the Experimental Section). Due to the formation of Mo—O bonds (to be demonstrated later), the oxygen atoms from atmosphere are introduced at the sulfur‐vacancy sites during their growth and transfer processes^26^ as displayed in **Figure** **2**a, which further affects the interfacial strain and the many‐body interactions in MoS_2_.^26^ The calculated optical spectra for monolayer‐MoS_2_ with and without the STO substrate are shown in Figure 2b,c. Electron–electron and electron–hole interaction parameters are incorporated in GW‐RPA (random‐phase approximation) and GW‐BSE (Bethe–Salpeter equation) calculations, respectively. Interestingly, an additional peak at ≈3.94 eV appears in the GW‐BSE spectrum for monolayer‐MoS_2_/STO in Figure 2b—comparable to the experimentally observed 3.83 eV peak in Figure 1e. Since it only appears in the GW‐BSE spectrum (with electron–hole interactions) and not in the GW‐RPA spectrum in Figure 2b,^12^ this is a resonant excitonic peak, such that only its resonant component is taken into account based on the Tamm–Dancoff approximation. Further comparison of GW‐BSE spectra with and without STO substrate in Figure 2c affirms that this excitonic peak is a result of the STO.

Figure 2d displays the temperature‐dependent absorption spectra of MoS_2_/STO (see Figure S9 in the Supporting Information for full temperature data). As temperature decreases, peak D sharpens and is redshifted. Recent studies have shown that excitonic effects in STO due to strong electron–hole interactions can be observed in its optical spectra.^12^ The strong temperature‐dependent excitonic feature in STO at ≈3.8 eV is also observed in Figure 2e and more conspicuously displayed in the inset (differential absorption: *Δα*(ω,*T*) = α(ω,*T*)−α(ω,350 K)) where there is similar trend of sharpening and redshifting with decreasing temperature. This feature has been attributed to Wannier‐like excitons in STO.^12^ The optical spectra of MoS_2_/STO and the corresponding spectra of STO at the same energy as well as the GW‐BSE calculation provide clear evidence that the excitonic wavefunctions of STO substrate extend into monolayer‐MoS_2_ through interfacial orbital hybridization.

**Figure 3:**
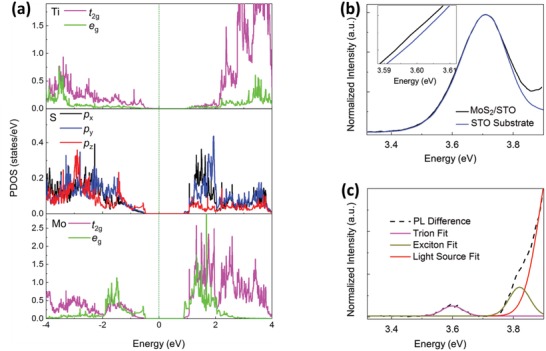
DOSs and high‐energy photoluminescence spectra. a) The DOSs of the MoS_2_ monolayer on the STO substrate projected on d‐orbital of Ti atoms, p‐orbital of S atoms, and d‐orbital of Mo atoms at the interface TiO_2_ sublayer. b) Normalized photoluminescence spectra of monolayer‐MoS_2_/STO and STO substrate at 77 K. Inset: Close‐up view to compare PL spectra of monolayer‐MoS_2_/STO and STO. c) Difference in photoluminescence spectra (dashed lines) along with peak fittings of excitons *D* and *D*′ in monolayer‐MoS_2_ at 77 K. Additional peak is included to account for the photoluminescence source.

Results from first‐principles calculations further confirm the presence of interfacial orbital hybridization. As shown in Figure 2a, the calculated bond length of interfacial Ti—S bond is ≈2.63 Å, close to that of covalent Ti—S bonds in TiS_2_. This implies an interfacial Ti—S orbital hybridization. A previous study also shows weak interfacial adsorption with interfacial orbital hybridization.^27^ Further calculated partial density of states (PDOS) analysis (**Figure** **3**a and Figure S1, Supporting Information) shows that p_z_‐orbitals of S‐atoms interact directly with Ti‐3d orbitals, leading to a significant orbital resonance between −4 and −0.5 eV. More specifically, the density of states calculation of the Ti and S atoms as displayed in Figure 3a shows greater and significantly more pronounced overlap between the S‐p_z_ orbitals and the t_2g_ orbitals belonging to Ti‐3d near the valence and conduction band edges. This larger overlap implies a stronger interaction between these two sets of aforementioned orbitals within this energy region. The energy level of the t_2g_‐orbitals belonging to Ti‐3d is lower than the e_g_‐orbitals and they are located closer to the Fermi level. This suggests a stronger interaction between the S‐p_z_ orbital and the Ti‐3d t_2g_ orbitals. This will be experimentally demonstrated in our X‐ray linear dichroism (XLD) absorption measurement as discussed later (c.f., Figure 5). Interfacial orbital hybridization enables the orbital that originally is only occupied by S p‐electrons, to be occupied by Ti d‐electrons. This allows for some high‐energy states in monolayer‐MoS_2_ to have a mixed character with STO. Thus, this results in the propagation of excitonic wavefunctions from STO into monolayer‐MoS_2_ via their interface. Previous studies have shown that high‐energy excitons have occurred in STO^12^ and they can propagate through the interfaces.^13^ Therefore, in the case of monolayer‐MoS_2_ on STO, we believe that the excitons are propagated from the STO substrate and they belong to the MoS_2_ layer. The substitution of O‐atoms in the sulfur vacancies of monolayer‐MoS_2_ will suppress the formation of high‐energy exciton. This is due to the reduction of Ti‐S hybridization at the MoS_2_/STO interface. To verify that the presence of the O substitution into the S vacancies in the MoS_2_ monolayer has an effect on the high‐energy exciton feature, the original MoS_2_/STO sample has been re‐measured under ex situ condition over an extended period and its absorption spectrum compared with the original spectrum. Figure S10 (Supporting Information) compares the absorption spectra of the previous MoS_2_/STO at 300 K and the ex situ spectrum in its current state. The absorption spectrum of the aged MoS_2_/STO sample shows an intensity reduction slightly below that of the original 300 K measurement. This result further indicates the continual O substitution that reduces the Ti‐S hybridization at the MoS_2_/STO interface. While there is an ageing process taking place in the MoS_2_/STO with O substitution into the S‐vacancies, the sample remains rather stable even under atmospheric condition.

Interestingly, the exciton peak splits into two peaks, D and D′, at low temperature (Figure 2d, ≈0.15 eV energy split at 77 K). These two exciton peaks have also been demonstrated using photoluminescence spectroscopy as discussed later. It has been reported that there is strong spin–orbit coupling in 2D‐TMDs,^7^ which has led to the split into excitons A and B (≈0.15 eV apart at 77 K). The split of exciton into D and D′ can also be attributed to the effect of spin–orbit coupling. Using the temperature‐dependent absorption linewidth of the observed peaks,^28^ binding energy (BE) of D and D′ is estimated to be (13.66 ± 1.64) meV and (14.48 ± 1.37) meV, respectively (details are provided in the Methods section of the Supporting Information). These estimated BEs are significantly smaller than those reported of low‐energy excitons A and B.^10^ BEs of D and D′ are further verified based on temperature‐dependent spectral‐weight analysis of the excitons where drastic spectral‐weight increases in the ≈200 K (≈17 meV) region are recorded (Figure S4, Supporting Information), signifying the dissociation of excitons into their individual charged constituents above their respective BEs. The weaker BEs may potentially impact the development of new low‐temperature photonic and optoelectronic devices and sensors.

### 2.3 Photoluminescence Spectroscopic Characterization to Verify the High‐Energy Excitonic Peaks

To further evaluate the excitonic effects brought about by the interfacial dynamics of monolayer‐MoS_2_/STO in UV–vis region, high‐energy PL study is conducted at various temperatures. Figure 3b compares the normalized PL data between MoS_2_/STO and STO at 77 K. Although both spectra are similar with a prominent excitonic signal that the STO substrate displays, marked differences are observed at the ≈3.60 eV (see figure inset) and ≈3.80 eV region. Besides, these spectral differences are consistently present with repeated measurements at different temperatures (Figure S5, Supporting Information). The spectral differences between monolayer‐MoS_2_/STO and STO are studied by a fitting analysis using two Gaussian lineshapes as displayed in Figure 3c (details are provided in the Methods section of the Supporting Information). Interestingly, the peak positions and widths of the lineshapes are consistent with the profiles of peaks D and D′ elucidated from spectroscopic ellipsometry data. Analytical results are strong indications pointing to the existence of high‐energy excitons. Despite monolayer‐MoS_2_ (≈1 nm) being much thinner than the STO substrate, the PL signal suggests an influential effect of the high‐energy interfacial excitonic responses on the radiative properties of monolayer‐MoS_2_.

### 2.4 Observation of Fermi‐Surface Feature at the MoS_2_/STO Heterointerface

**Figure 4:**
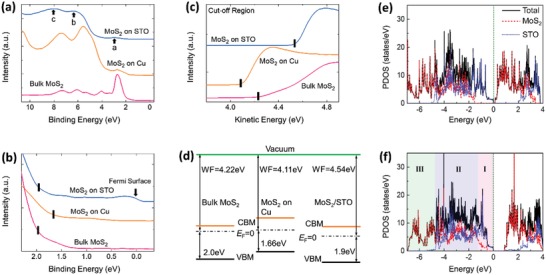
Synchrotron‐based photoemission spectra of MoS_2_/STO, MoS_2_ on copper, and bulk MoS_2_. a) Valence band spectra up to 10.7 eV. b) Close‐up of valence band spectra reveals the formation of a Fermi‐surface feature near 0 eV binding energy. The ticks indicate the position of the valence band minimum location. c) Work‐function measurements. The ticks indicate the apparent cutoff energy positions of the work function. d) Schematic of the band alignments derived from photoemission measurements showing work function (WF), Fermi level (*E*_F_), valence band maximum location determined from valence band spectra (VBM*), and conduction band minimum (CBM). e,f) The total and projected DOSs of MoS_2_ monolayer on the STO substrate at large spacing distance ((e) 14 Å) and the optimized spacing distance ((f) 2.58 Å). The Fermi levels are aligned to the vacuum level and shifted to 0 eV. Regions I, II, and III are related to peaks a, b, and c in (a), respectively.

**Figure** **4**a compares the valence band spectra up to 10.7 eV between MoS_2_/STO, MoS_2_ on Cu, and bulk MoS_2_ using synchrotron‐based photoemission spectroscopy (PES). According to previous photoemission spectroscopy studies,^29^, ^30^ peak a is attributed to the hybridization between Mo4d_z2_ and S3p_z_ (and O2p_z_) orbital; peak b is ascribed to O2p and Ti3d bands intermixed with MoS_2_ p‐d valence band; peak c is ascribed to the p‐d covalent bonding band between O and Mo‐atoms intermixed with MoS_2_ p‐d valence band.

Interestingly, an anomalous Fermi‐surface feature (arrow in Figure 4b) is observed at the MoS_2_/STO interface shown in the close‐up of valence band spectrum. This is consistent with the optical result in Figure 1e showing the presence of carriers in MoS_2_/STO. It implies that the excitonic effect propagation from the STO substrate also has an appreciable impact on the weak electronic correlations in the MoS_2_ monolayer. The increased weak electronic correlations in MoS_2_ may affect the Fermi surface instability that is induced by symmetry breaking of the MoS_2_ film that manifests itself in the form of the Fermi‐surface feature. This feature is not observed for MoS_2_ films on other substrates. The MoS_2_/STO valence band maximum (VBM*, location determined from valence band spectra as indicated by the ticks; details are provided in the Supporting Information) is shifted to a higher BE as compared to MoS_2_/Cu but is lower than that of bulk‐MoS_2_. Figure 4c shows a significant increase in work function (denoted by the ticks) of monolayer‐MoS_2_/STO compared to bulk‐MoS_2_ and MoS_2_/Cu. The shifts in work function and VBM* in MoS_2_/STO are possible effects of interfacial orbital hybridization, charge transfer, and lattice distortion. As noted in Figure 4c, the work function is substrate dependent. Hence, it suggests the possibility of tuning the physical and chemical properties of monolayer‐MoS_2_. Since the peak of bulk MoS_2_ is broad due to point defects, surface roughness, impurities, and vacancies, to ensure consistency, the work function is derived by intersecting the extrapolated leading edge with the background baseline (details are provided in the Supporting Information). Schematics of the band offsets are depicted in Figure 4d based on photoemission data. This increased mobile electrons and tunable work function in MoS_2_/STO can be harnessed to increase the efficiency of nanoelectronic devices.^8^ The presence of these free charge carriers may enhance the electron–electron screening, which yields to the spectral weight transfer and may affect the resonant excitons.^12^ However, we note that how the free carriers affect the resonant exciton deserves its own study and would be an interesting problem to be addressed in future.

### 2.5 Analysis of Interfacial Hybridization at the MoS_2_/STO Heterointerface

**Figure 5:**
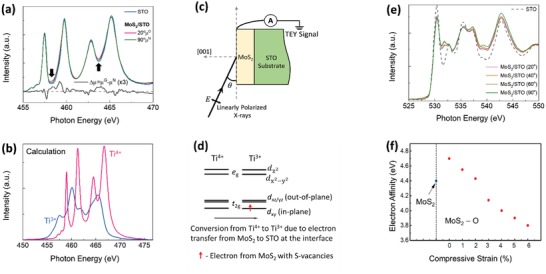
Characterizing Ti and O valence state for MoS_2_/STO and bulk STO. a) Polarization‐dependent Ti L‐edges XAS results of MoS_2_/STO and bulk STO. b) Independent multiplet calculations of the Ti^3+^ and Ti^4+^ states. c) Schematic picture of the experimental configurations for XLD measurements. d) Energy diagram of crystal field splitting and 3d orbital degeneracy and its reconstruction at the interface with mixed valence Ti^3+^ and Ti^4+^ states. e) Polarization‐dependent O K‐edge XAS of MoS_2_/STO heterostructure and bulk STO. f) The calculated electron affinity of pristine monolayer‐MoS_2_ and monolayer‐MoS_2_ with an oxygen atom at the sulfur site. Biaxial compressive strain is applied on monolayer‐MoS_2_ due to the substitution oxygen atom.

To experimentally validate the occurrence of interfacial hybridization and charge transfer at the MoS_2_/STO heterointerface, room temperature X‐ray absorption spectroscopy (XAS) measurements were performed. It is noted that monolayer‐MoS_2_ is too thin to yield decent Mo and S XAS signals for effective characterization. Therefore, we turn instead to the characterization of the Ti L and the O K‐edges in the XAS measurements. This indirect but effective characterization methodology that bypasses the restriction of monolayer‐MoS_2_ being too thin serves to provide essential information on the formation of the Mo—O bonds. The Ti L and the O K‐edges of the bulk STO are further used as a reference to confirm the formation of Mo—O bonds that will be discussed subsequently. Polarization‐dependent Ti L‐edges XAS for MoS_2_/STO and bulk‐STO are shown in **Figure** **5**a. Comparisons are made with independent multiplet calculations of Ti^4+^ and Ti^3+^ in Figure 5b reflecting Ti2p_j_→Ti3d transitions. The spectra show four features attributed to spin–orbit‐coupling splitting (2p_3/2_ and 2p_1/2_) and crystal‐field splitting (e_g_ and t_2g_). With the multiplet calculations as references, the increased intensities at ≈458 and ≈464.5 eV (arrows in Figure 5a) correspond to the rise in Ti^3+^ concentration. This clearly indicates an electron transfer from monolayer‐MoS_2_ to STO, with the main contribution coming from the interface. As total electron yield (TEY) model is sensitive to few monolayers. This indicates the presence of Ti^3+^ ions at the interface and the onset of charge transfer. This is consistent with the estimated effective electron number (≈0.01 per MoS_2_ unit formula) from ellipsometry measurement. This electron‐transfer process is further supported by polarization‐dependent Ti L‐edge XLD shown at the bottom of Figure 5a, which shows the intensity difference between the spectra measured at 20° (out‐of‐plane) and 90° (in‐plane) polarizations (Figure 5c). Smaller absorption for in‐plane polarization spectra suggests more out‐of‐plane empty states in the 3d t_2g_ band (a higher occupancy of in‐plane orbitals). The spectra difference (*I*(**E**||*c*)‐*I*(**E**||*a*), linear dichroism) implies a preferential occupancy of the 3d_xy_ orbital. It demonstrates the presence of mixed Ti^3+/4+^. The positive integrated XLD implies a larger out‐of‐plane absorption—more out‐of‐plane (d_xz/yz_) empty states while the transferred electrons are in the in‐plane orbitals (d_xy_) (Figure 5d).

Polarization‐dependent O K‐edge XAS of the MoS_2_/STO heterostructure and bulk‐STO are shown in Figure 5e. The O K‐edge spectra reveal transitions from O1s to unoccupied O2p states, which hybridize with metallic states. The peak at ≈530.4 eV corresponds to the O2p‐Ti3dt_2g_ orbital hybridization while the O2p‐Ti3de_g_ orbital hybridization is located at ≈532.8 eV.^31^ While the O2p‐Ti3de_g_ peak intensity remains unchanged, the O2p‐Ti3dt_2g_ peak of MoS_2_/STO falls below that of bulk‐STO. This can be attributed to the transferred electrons partially occupying the Ti3dt_2g_ states. Besides, Ti3dt_2g_‐S3p hybridization at MoS_2_/STO interface reduces the O2p‐Ti3dt_2g_ hybridization at STO surface. The features above 534 eV are attributed to O2p hybridizing with higher energy metal states (e.g., Ti‐s,p and Sr‐s,p,d).

To further analyze the interfacial charge transfer and the electronic structure in MoS_2_/STO, it needs to combine both Ti L_3,2_ and O K‐edges XAS spectra. In Figure 5e, it is shown that the change of XAS yield at O K‐edge between bulk STO and MoS_2_/STO, particularly at 530–532 eV (O2p‐Ti3d hybridization states), is significant. While the change of XAS yield at Ti L_3,2_ edges between bulk STO and MoS_2_/STO in Figure 5a is rather small. These observations reveal that the charges at the interface are mainly delocalized in the Ti‐O hybridization states and only a small fraction is localized in Ti atoms.

It is interesting to observe an unprecedented polarization‐dependent hybridization peak at ≈531.9 eV not found in bulk STO. Based on previous studies on molybdenum oxides,^32^ a similar energy position of this peak suggests that it can be attributed to O2p‐Mo4d hybridized states. In Figure 5e, while the Ti3dt_2g_‐O2p (≈530.4 eV) and Ti3de_g_‐O2p (≈532.8 eV) peaks are nearly polarization independent, the new O2p‐Mo4d peak (≈531.9 eV) is polarization dependent. The spectra for the in‐plane (90°) and out‐of‐plane differ due to the crystallinity of Mo—O.^32^ Thus, there is strong anisotropy in the electronic properties. Moreover, the absorption intensity measured at 40° is slightly higher than that of 20° and 60°. This suggests that Mo—O hybridization bonds are mostly at 40°, which is close to the Mo—S bond angle of ≈40° for 2H‐MoS_2_ reported in a previous study.^33^ Therefore, we postulate that some of the sulfur vacancies in CVD‐fabricated monolayer‐MoS_2_ are partially occupied by the O‐atoms (Figures 1e and 2a) during its transfer process,^26^ thereby forming Mo—O covalent bonds.

As previously reported,^34^ the remaining sulfur vacancies in 2D‐MoS_2_ act as electron donors, which induce localized states in the band gap. There is no formation of a Fermi surface in free‐standing MoS_2_ due to the localized nature of the electrons. It has been demonstrated in 2D‐heterostructure that charge transfer across the interface is controlled by the competition between the electron‐affinity mismatch and the level of film‐substrate hybridization.^35^ Based on our computational study, both oxygen occupation at sulfur‐vacancy sites and interfacial compressive strain lead to a decrease in electron affinity of monolayer‐MoS_2_ (Figure 5f). When the electron affinity of monolayer‐MoS_2_ falls below that of STO (3.9 eV^36^), the electron‐affinity mismatch and the excess localized electrons (sulfur vacancies) increase the likelihood of electron transfer from monolayer‐MoS_2_ to STO. The competing properties between electron‐affinity mismatch and interfacial hybridization result in the presence of interfacial mobile electrons.

To further substantiate the agreement between experimental and theoretical studies, we perform further first‐principles studies involving monolayer‐MoS_2_ in two different interfacial atomic positions on STO (both the MoS_2_ monolayers being shifted from the most stable position; details are provided in the Supporting Information), which are shown in Figures S7 and S8 (Supporting Information). This is to provide further verification that interfacial hybridization and the formation of the new high‐energy exciton still take place even with the stacking of MoS_2_ and STO in different configurations as long as there are strong interfacial interactions between the MoS_2_ and STO substrate. It also shows that the onset of the new exciton is attributed to the unique interfacial hybridization and it is weakly correlated to the stacking configuration between the TMDs and the STO substrate.

## 3 Conclusion

By unraveling the complex spin–orbit–charge–lattice coupling at MoS_2_/STO heterointerface, it provides a deeper insight into the mechanisms leading to the new excitonic and electronic effects. The study of the interfacial phenomena is based on a comprehensive methodology that comprises temperature‐dependent spectroscopic ellipsometry, photoluminescence, X‐ray absorption and photoemission spectroscopies, and first‐principles calculations. Detailed study of these anomalous excitons uncovers new insights into existing theories, which is related to the interband transitions, and underlines many of the intriguing physical properties, thereby exposing them to further examination.

## 4 Experimental Section

*Sample Preparation*: The monolayer‐MoS_2_ was synthesized on a sapphire (Al_2_O_3_) surface by the CVD method using MoO_3_ and S powders as the reactants.^17^ The monolayer‐MoS_2_ was then transferred to TiO_2_‐terminated (001)‐STO substrate and Cu foil using polymethylmethacrylate. The TiO_2_‐terminated STO substrate was obtained through ultrasonic treating in buffered hydrofluoric acid solution and annealing in a furnace at 950 °C for 1.5 h in air with a ramping up rate of 5 °C min^−1^ and ramping down rate of 3 °C min^−1^. In this study, the TiO_2_‐terminated STO substrate was chosen because the 3d electrons of the transition metal Ti‐atoms play an important role in generating new and exotic properties in both the STO bulk materials and in the formation of other heterostructure systems.^37^, ^38^ Since STO was annealed in atmospheric pressure at high temperature, it was difficult to create oxygen vacancies inside. The PL and Raman measurements showed that the monolayer‐MoS_2_ was of high quality before and after the chemical transfer process. These experiments confirmed that the samples retained their high quality even after the transfer. Besides, the method of chemical transfer of monolayer‐MoS_2_ was preferred over the direct synthesis of MoS_2_ on SrTiO_3_ using the CVD process as the latter technique would introduce oxygen vacancies in the SrTiO_3_ substrate at high temperature in vacuum. It was demonstrated that sulfur vacancies were inevitably present in CVD‐fabricated monolayer‐MoS_2_ and that they were further generated during the transfer processes.^26^, ^34^ The O‐K edge spectra from the XAS data showed the presence of the Mo—O bond in the MoS_2_/STO heterostructure. This was a clear demonstration of O substitution into S‐vacancies in our monolayer‐MoS_2_ samples.

*Spectroscopic Ellipsometry Measurements and Absorption Coefficient*: A J. A. Woollam Co., Inc spectroscopic ellipsometer with photon energy of 0.6–4.5 eV was used to measure the ellipsometry parameters *Ψ* (the ratio between the amplitude of p and s‐polarized reflected light) and *Δ* (the phase difference between p and s‐polarized reflected light) in a high vacuum chamber with a base pressure of 1 × 10^−9^ mbar. The substrate layers (bulk SrTiO_3_ or Al_2_O_3_) were also measured under the same conditions. The absorption coefficient, α, of MoS_2_ monolayer was extracted from parameters *Ψ* and *Δ* utilizing an air/MoS_2_/STO (or Al_2_O_3_) multilayer model, where monolayer‐MoS_2_ consisted of a homogeneously uniform medium^14^, ^23^ and a composite heterointerface component.

*Effective Electron Number*: Taking into consideration our optical data, by analyzing the optical conductivity spectrum of MoS_2_/STO at room temperature,^22^, ^23^ the estimated effective electron number per unit cell volume of MoS2 is defined below as a form of the spectral weight up to an energy

(1) $$N_{\mathrm{eff}}\left(\omega \right)=\frac{2m_{0}V}{\pi e^{2}}\;\int_{0}^{\omega }\sigma \left(\omega \right)d\omega$$

where *m*_0_ is taken as the free‐electron mass and *V* is the unit cell volume *V* = 0.054 nm^3^ of monolayer‐MoS_2_. The *N*_eff_(ω) is proportional to the number of electrons involved in the optical excitations up to ω. The optical conductivity at energy between 0 eV (estimated as 0) and 0.6 eV was estimated using a linear interpolation. The estimated effective number of carriers *N*_eff_ (1.5 eV), calculated from the optical conductivity spectra at energy range up to 1.5 eV,^22^, ^23^ standed at about 0.01.

*X‐Ray Absorption Spectroscopy (XAS)*: The O *K*‐edge absorption spectra in the energy range 520–580 eV and Ti L‐edge absorption spectra in the energy range 450–490 eV were obtained using linearly polarized X‐ray absorption spectroscopy from the Surface, Interface and Nanostructure Science (SINS) beamline at Singapore Synchrotron Light Source (SSLS), using a TEY detection method. The incidence angle (90‐θ) of X‐rays refers to the normal of the sample surface, which was varied by rotating the polar angle of the sample. The spectra were normalized to the integrated intensity between 565 and 580 eV for O1s spectra and between 475 and 490 eV for Ti2p spectra after subtracting an energy‐independent background.

*X‐Ray Linear Dichroism (XLD) Measurements*: For Ti, the 3d_xy_ and 3d_xz/yz_ orbitals had lobes pointing parallel and perpendicular to the *ab‐*plane, respectively. XLD could probe the occupancy of the 3d_xy_ and 3d_xz/yz_ orbitals using linearly polarized light aligned to the out‐of‐plane (grazing incidence 20°, **E**∼||*c*) and in‐plane (normal incidence 90°, **E**||*a*) directions (Figure 5c).

*Synchrotron‐Based Photoemission Spectroscopy (PES)*: The room temperature photoemission data were taken in an ultrahigh vacuum chamber with a base pressure of 1 × 10^−10^ mbar at the SINS beamline of SSLS. The photon energy of 60 eV was used to probe the valence band spectra. The work function was measured using 60 eV photon energy with a −7 V applied bias. The spectra were collected at normal emission using a VG Scienta R4000 analyzer and normalized by photon current. The binding energy is referred to the Fermi level of a sputter‐cleaned gold foil in electrical contact with the sample.

*Atomic Multiplet Calculations*: The simulations were performed using CTM4XAS software with octahedral (*O*_h_) crystal field 10 *D*_q_ = 1.85 eV, Hubbard value *U*_dd_ = 1.7 eV, and charge‐transfer energy *Δ* = 1.2 eV. The Slater integrals were taken to be 80% of the Hartree–Fock values, which represent the atomic values.^39^ Relative to the Ti^4+^‐multiplet states, calculations showed an overall spectral shift of the Ti^3+^‐multiplet states to lower photon energies (Figure 5b).

*Interfacial Model of MoS_2_/STO*: For the interface structure of monolayer‐MoS_2_ on STO (001), (√3 × 2) MoS_2_ supercell was placed on the (√2 × √2) STO (001) substrate with six atomic layers, in which tensile strain (about 14.2% tensile strain on the *b*‐direction) was applied on the STO as the electronic properties of monolayer‐MoS_2_ are sensitive to the external strain. The differential charge density was visualized by an isosurface value of 1.0 × 10^−3^ e Å^−3^. For the perfect monolayer‐MoS_2_ on STO (001) substrate, the interaction between them was found to be weak as the calculated adsorption energy is 37 meV Å^−2^ and layer spacing is about 2.62 Å, similar to that of monolayer‐MoS_2_ on the HfO_2_ or graphene on the STO substrate.^13^ It was noted that the shortest distance between Mo and O atoms was about 4.5 Å, much larger than potential bonding range. As orbital hybridization between Mo4d and O2p was characterized by X‐ray adsorption spectroscopy, which as suggested might be contributed by the incorporation of oxygen atoms in monolayer‐MoS_2_ during the transfer process. Thus, O defects were considered in MoS_2_ monolayer by substituting an S atom in the interface structure, which was related to surface oxygen density of 1/8. The Mo—O bond length is shorter than that of Mo—S bond. Hence, compared to the perfect monolayer‐MoS_2_, local compressive strain was expected in monolayer‐MoS_2_ with such O‐defects—consistent with Raman and PL data (Figure 1b,c). The presence of O‐defects in monolayer‐MoS_2_ slightly enhanced the interfacial interaction between MoS_2_ and STO, as supported by the increased adsorption energy (≈44 meV Å^−2^) and shorter interfacial spacing (2.58 Å). It was noted that in experiment the oxygen concentration in MoS_2_ might not be so high. Due to highly demanding GW‐BSE calculations, a lager supercell could not be used, but it is thought that the physics discussed here would not be affected significantly by this relatively small interface supercell. The adsorption of monolayer MoS_2_ on the STO was weak as the calculated adsorption energy was about 44 meV Å^−1^. The weak interfacial interaction is also confirmed by the unnoticeable interfacial charge transfer in Figure 2a and also Bader charge analysis.

*First‐Principles Calculations*: All calculations were carried out by using density‐functional theory based the Vienna Ab initio Simulation Package (VASP) with the Perdew–Burke–Ernzerhof format exchange‐correlation functional and the projector augmented wave potentials.^40^, ^41^, ^42^ The energy cutoff for the electronic plane‐wave expansion was set to 500 eV. A 12 × 9 × 1 Monkhorst–Pack‐based *k*‐point mesh was used for sampling the first Brillouin zone.^43^ In order to minimize the artificial interaction between repeated slabs imposed by the periodic boundary condition, a vacuum layer with 25 Å thickness was applied normal to the surface of MoS_2_ monolayer. Van der Waals correction to the dispersion energy was included by using Grimme's DFT‐D3 method.^44^ Moreover, an effective Hubbard *U* = 4.0 eV was applied to d‐orbital of Ti atoms in order to take into account the orbital‐dependent Coulomb and exchange interaction. Besides, dipole correction was included in the calculations to minimize the errors introduced by the periodic boundary conditions. In all calculations, the energy and force on each atom were converged to be smaller than 1.0 × 10^−6^ eV and 0.01 eV Å^−1^, respectively. The bottom two layers of STO were fixed during the structural relaxation.

Due to the large excitonic effects in monolayer‐MoS_2_, a more advanced GW‐BSE method was used to calculate the optical spectra of monolayer‐MoS_2_/STO interface and monolayer‐MoS_2_ structures, where the many‐body effects were well described. In GW‐BSE calculations, more than 300 empty bands and a plane‐wave cutoff of 200 eV were included for the summation of the imaginary dielectric function. A total of 30 highest valence bands and 30 lowest conduction bands were included as a basis for the excitonic eigenstates. With the presence of the STO substrate, the electron–hole interaction was partially screened, leading to a binding energy estimated at ≈0.52 eV by comparing the energy position difference between the first transition peaks from the GW‐RPA and GW‐BSE calculations in Figure 2b. It was noted that the peak position of the new exciton was about 0.11 eV higher that that observed in the experiment, which might be due to larger tensile strain applied on STO in the calculations than that in experiment. Weak Drude response could be observed in the experiment (see Figures 1e and 2d), which was absent in the calculation. This difference resulted from the fact that in the experiment the S vacancies in the MoS_2_ monolayer were not fully compensated by the O atoms, making the MoS_2_ monolayer electron‐doped. While in the simulations, due to highly demanding GW‐BSE calculation, only a relatively small size of MoS_2_/STO supercell was considered, in which one S vacancy was compensated by an O atom, the whole system was still semiconducting. Split peaks A and B in Figure 1d were attributed to the strong spin–orbit coupling, which were not considered in the calculation.

## Conflict of Interest

The authors declare no conflict of interest.

## Supporting information

Supplementary
