# Supplementary material for: Modulation of New Excitons in Transition Metal Dichalcogenide‐Perovskite Oxide System
Source: Adv Sci (Weinh). 2019 Apr 29;6(12):1900446. doi: 10.1002/advs.201900446 (PMC6662271; doi:10.1002/advs.201900446)
Supplement: Supplementary file 1 — Supplementary [file ADVS-6-1900446-s001.pdf]

Copyright WILEY-VCH Verlag GmbH & Co. KGaA, 69469 Weinheim, Germany, 2019.

Supporting Information

for *Adv. Sci.,* DOI: 10.1002/advs.201900446

Modulation of New Excitons in Transition Metal Dichalcogenide-Perovskite Oxide System

Xinmao Yin, Ming Yang, Chi Sin Tang, Qixing Wang, Lei Xu, Jing Wu, Paolo Emilio Trevisanutto, Shengwei Zeng, Xin Yu Chin, Teguh Citra Asmara, Yuan Ping Feng, Ariando Ariando, Manish Chhowalla, Shi Jie Wang, Wenjing Zhang,* Andrivo Rusydi,* and Andrew T. S. Wee**

Copyright WILEY-VCH Verlag GmbH & Co. KGaA, 69469 Weinheim, Germany, 2019.

Supporting Information

Modulation of new excitons in transition metal dichalcogenide-perovskite oxide system

Xinmao Yin, Ming Yang, Chi Sin Tang, Qixing Wang, Lei Xu, Jing Wu, Paolo Emilio Trevisanutto, Shengwei Zeng, Xin Yu Chin, Teguh Citra Asmara, Yuan Ping Feng, Ariando, Manish Chhowalla, Shi Jie Wang, Wenjing Zhang*, Andrivo Rusydi*, Andrew T. S. Wee**

*Correspondence to: sj-wang@imre.a-star.edu.sg (S.J.W), wjzhang@szu.edu.cn (W.Z), phyandri@nus.edu.sg (A.R), phyweets@nus.edu.sg (A.T.S.W.).

Supplementary Figures

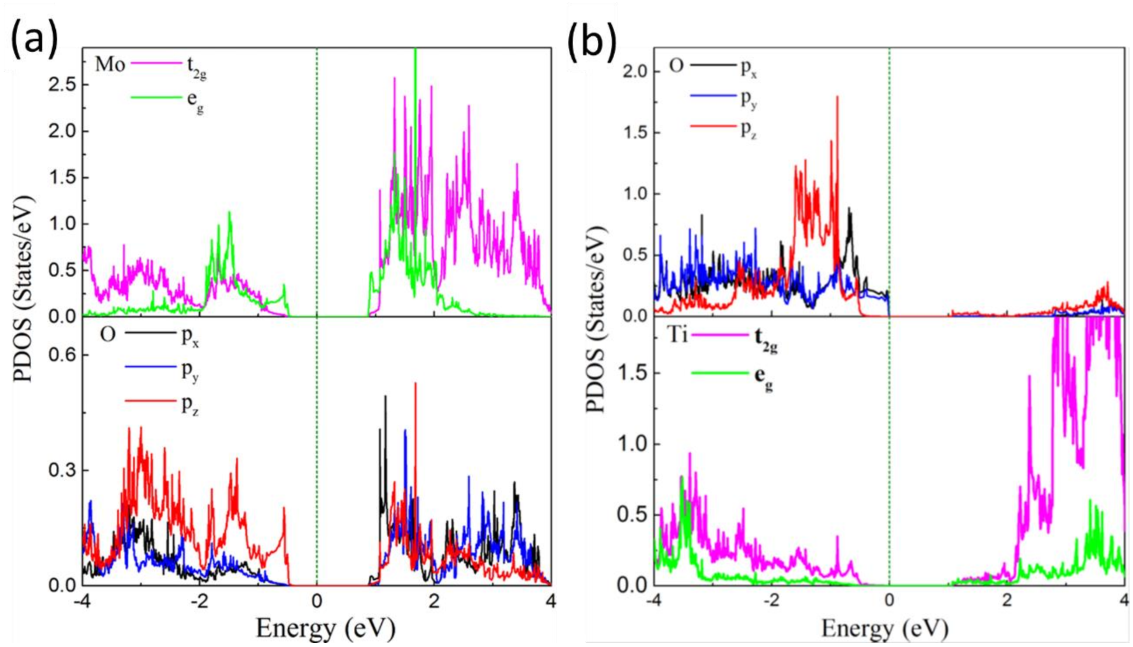

Figure S1. Partial Density of States (PDOS). **(a)** PDOS of Mo-atom and its neighbouring O-atom. (b**)** PDOS of interfacial Ti-atom and its neighbouring O-atom.

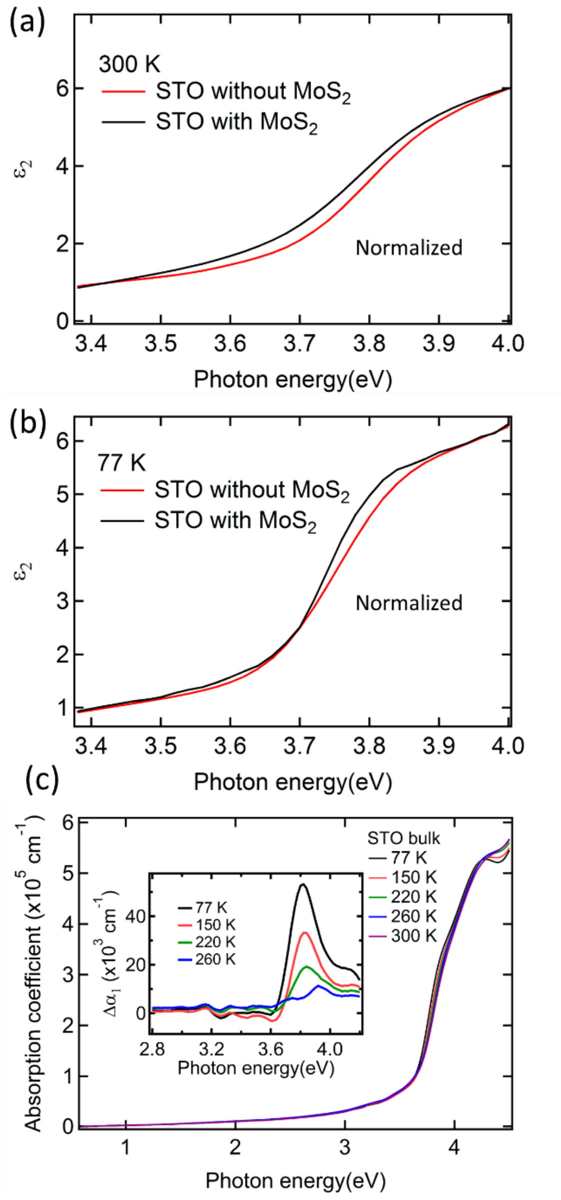

Figure S2. (a,b) Extracted ε_2 fitting for STO with and without the monolayer-MoS_2 at around 3.8eV at room temperature and low temperature (77K). (c) $\alpha_1(\omega)$ from 0.4 to 4.5eV as a function of temperature of STO bulk without MoS_2. Inset: differential absorption coefficient, $\Delta\alpha_1(\omega,T)=\alpha_1(\omega,T)-\alpha_1(\omega,300K)$.

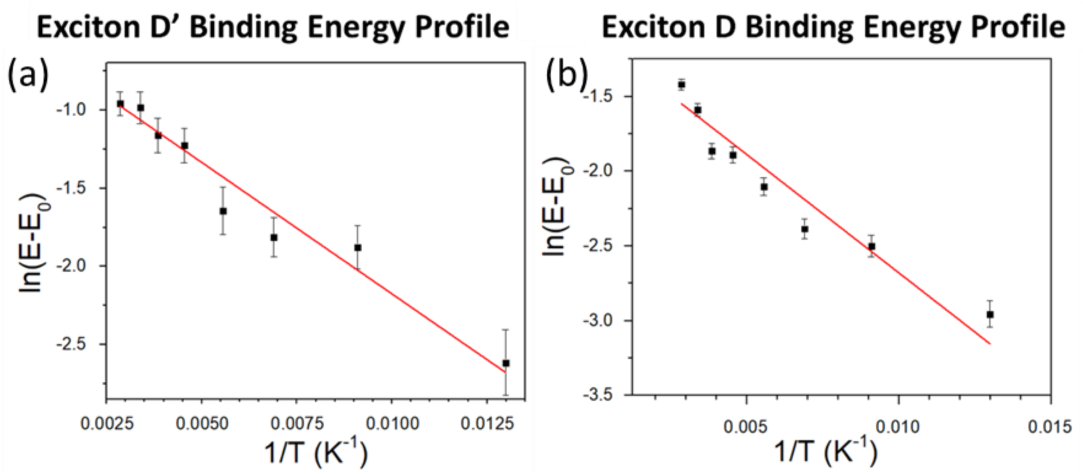

Figure S3. Estimation of binding energies for the high-energy excitons D and D'. Plot of excitonic linewidth with respect to the inverse of temperature yields a binding energy (a) of (14.48±1.37)meV for exciton D' and (b) (13.66±1.64)meV for exciton D .

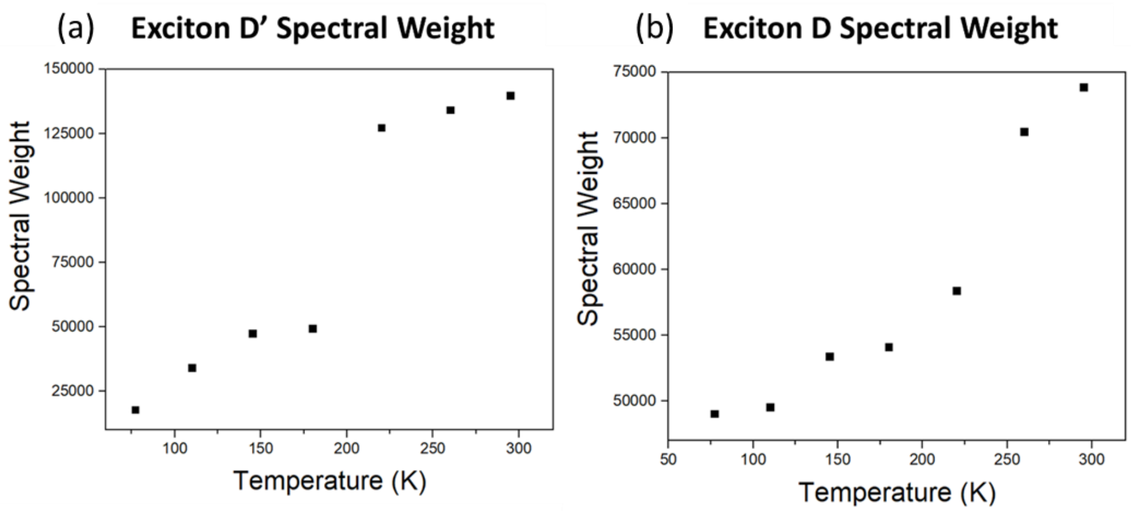

Figure S4. Spectral weight of excitons D and D' as functions of temperature. Plot of estimated spectral weight of exciton (a) D' and (b) D along with their evolution with respect to temperature.

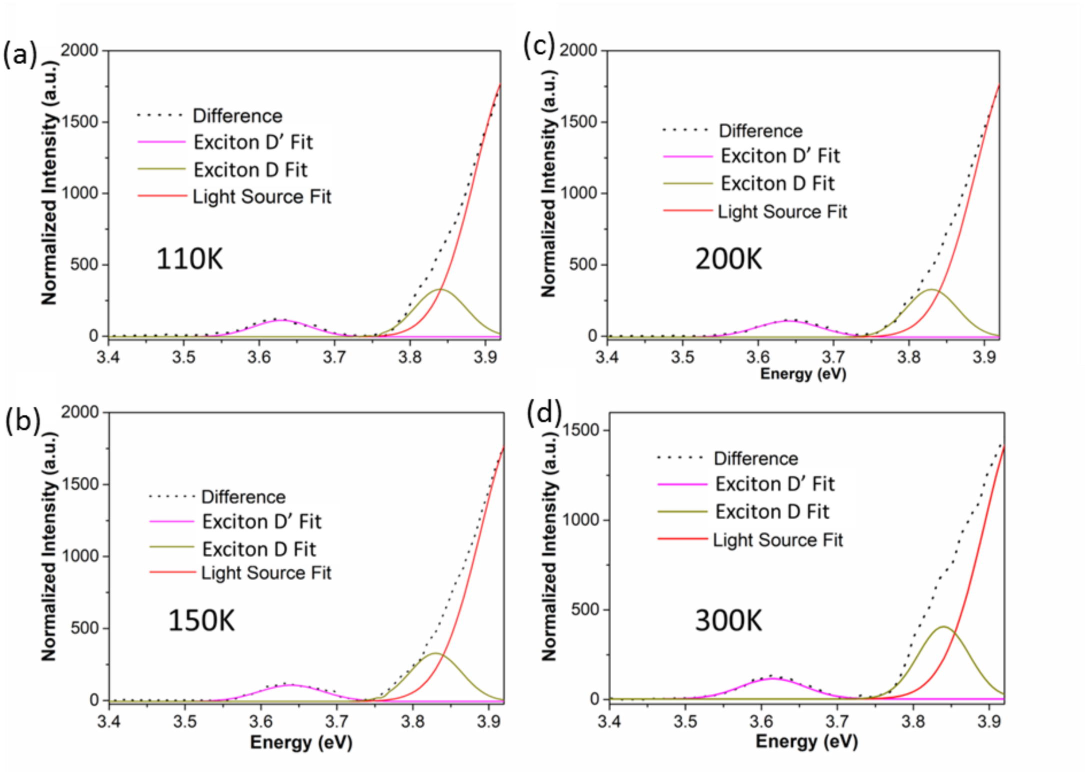

Figure S5. Photoluminescence spectra differences as functions of temperature. Differences in normalized photoluminescence spectra between monolayer-MoS_2/STO and STO substrate at (a) 110K (b) 150K (c) 200K (d) 300K along with peak fittings of excitons D and D' in monolayer-MoS_2. Note that an additional peak is incorporated to account for the PL light source.

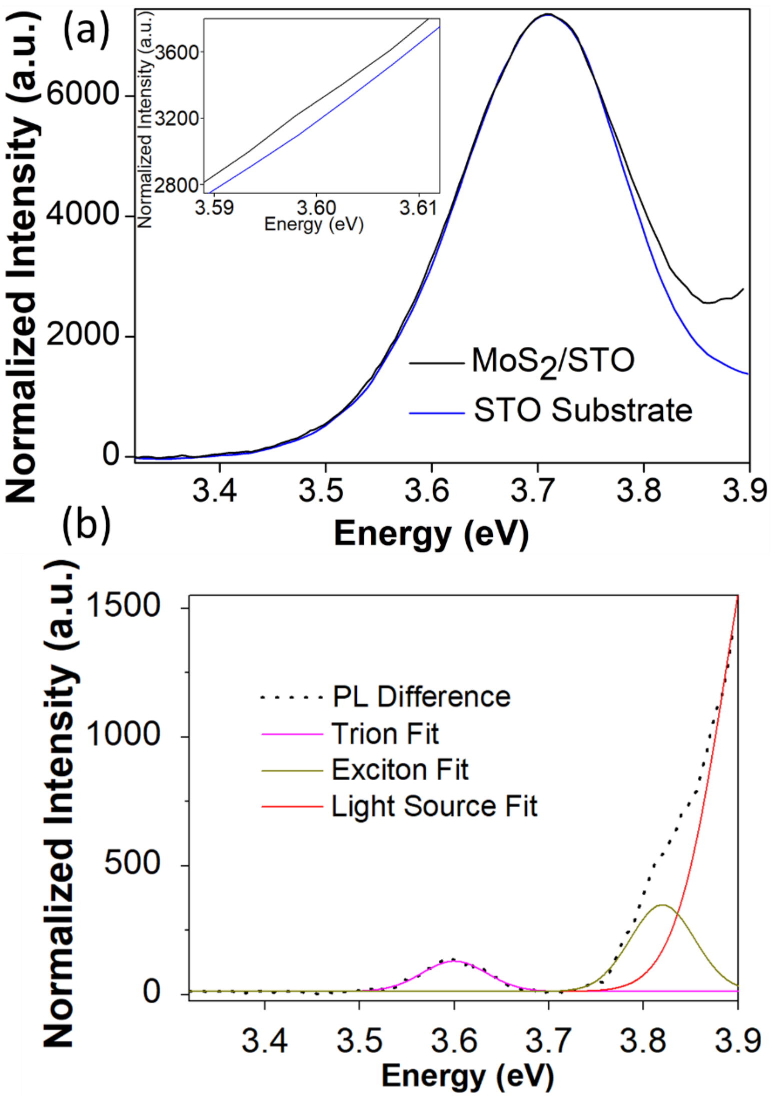

Figure S6. Photoluminescence spectra differences at 77K. (a) Normalized photoluminescence spectra of monolayer-MoS_2/STO and STO substrate at 77K. Inset: Close-up view to compare PL spectra of monolayer-MoS_2/STO and STO. (b) Difference in photoluminescence spectra (dashed lines) along with peak fittings of excitons D and D’ in monolayer-MoS_2 at 77K. Additional peak is included to account for the PL source.

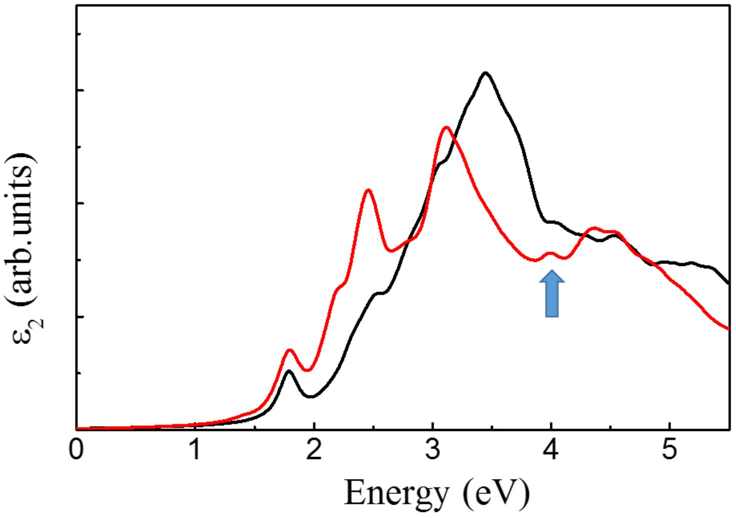

Figure S7. GW-BSE-calculated imaginary part of Dielectric function, ε_2, of monolayer-MoS2 on STO with two different configurations (with both MoS_2 monolayers configurations being shifted from the most stable position).

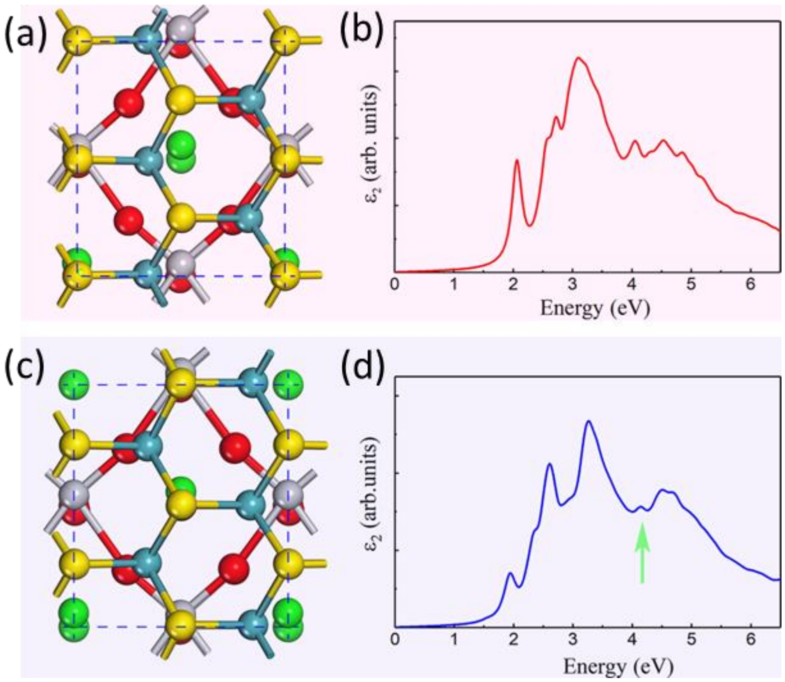

Figure S8. (a) The most stable configuration of MoS_2 monolayer on the STO (001) substrate and (b) the corresponding GW-BSE optical spectra. (c) A metal-stable configuration of MoS_2 monolayer on the STO (001) substrate and (d) the corresponding GW-BSE optical spectra. Note that the first exciton peak is shifted to experimental value.

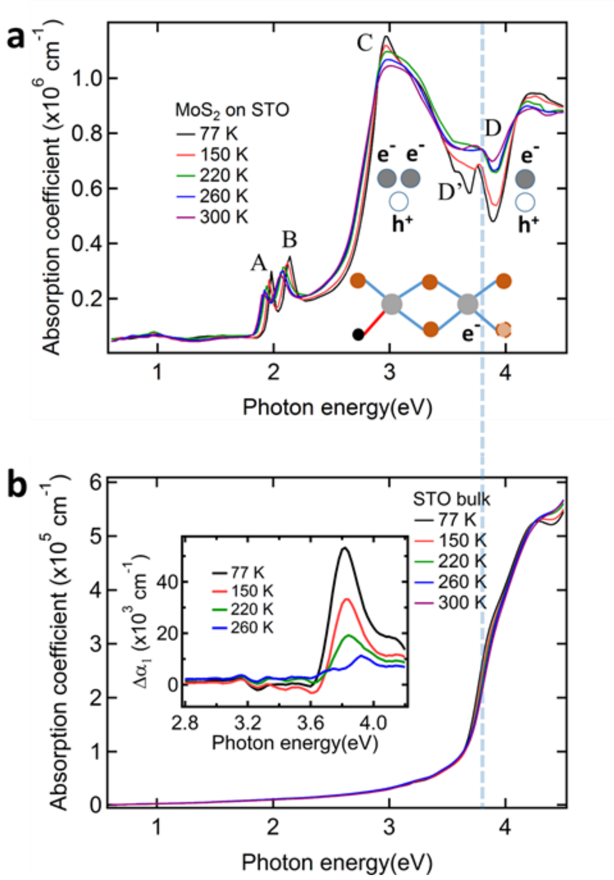

Figure S9. $\alpha(\omega)$ from 0.4 to 4.5eV as a function of temperature of (a) MoS_2/STO and (b) STO bulk with dashed line serving as visual guide. (Inset: differential absorption coefficient, $\Delta\alpha(\omega,T)=\alpha(\omega,T)-\alpha(\omega,300K)$).

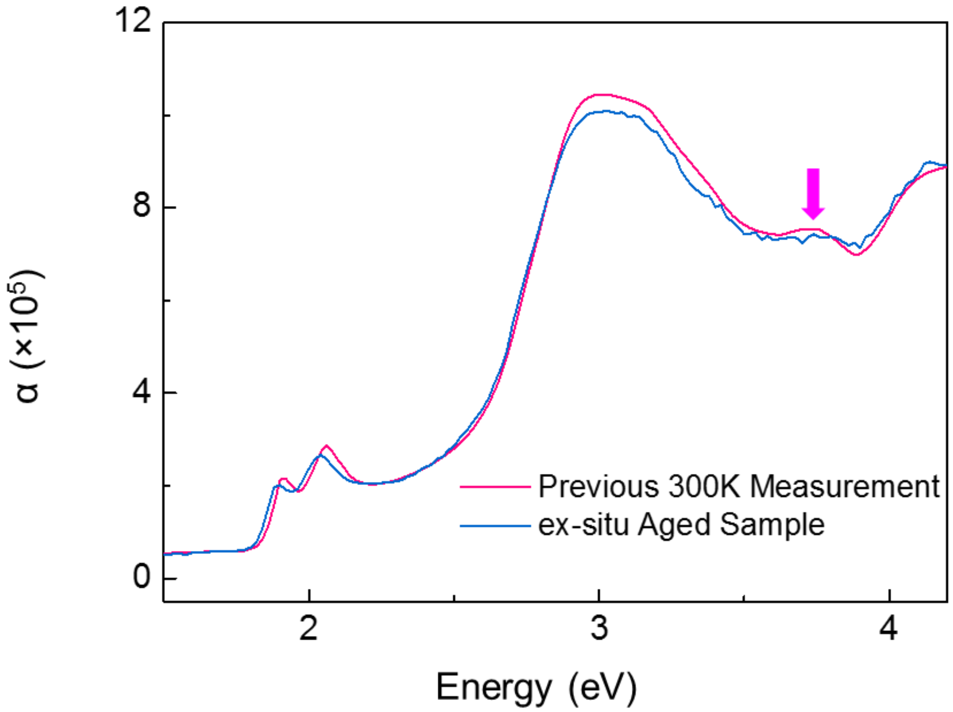

Figure S10. Comparing the Absorption coefficient, α, of the original MoS_2/STO measurement at 300K and after it has been aged.

Supplementary Methods

A new direction for interfacial effects: STO as a substrate

Firstly, from the perspective of the possible interaction between MoS_2 and potential substrates, the key reason STO is chosen as an ideal substrate because its band gap is only slightly higher than that of monolayer-MoS_2. The comparable band gaps of MoS_2 and STO results in the presence of band overlap at the Fermi level of the MoS2/STO interface. The unique compatibility of these two materials can increase the possibility of interfacial hybridization. Such interaction between a 2D-material and an oxide substrate

is absent from MoS_2 on other oxide substrates such as Al_2O_3, SiO_2 or even perovskites like the $LaAlO_3$.

According to the DFT band alignment diagram (Fig. 2(f)), there is a great likelihood that the S-orbitals of the MoS_2 monolayer hybridized with Ti-orbitals of the STO substrate. Hence, the STO substrate is treated to be TiO_2-terminated (001)-STO to provide the means for greater interaction between the monolayer-MoS_2 and STO. Through our comprehensive experimental and computational studies, we demonstrate the presence of interfacial interactions that beyond that of van der Waals interaction. It shows that 2D-TMD have a strong interaction with a suitable TMO substrate where it has a specifically specified surface termination. Our current study opens a new perspective and creates novel opportunities in the study of other TMD/TMO heterostructures. Such interfacial hybridization can possibly affect other interesting phenomena such as superconductivity, ferromagnetic effects, etc in different heterostructure. Hence, the investigation of this interfacial phenomenon in our manuscript has wide interest such that the large scientific community working on heterointerfaces can benefit from our scientific motivation introduced here.

From the perspective of the excitonic properties in STO, we note that STO in its bulk form and recently, in heterostructure systems with other oxides, have shown exotic phenomena such as superconductivity, magnetisms, metal-insulator transitions and a two-dimensional electron gas. It is worth mentioning that for more than 45 years, the existence of excitonic effects had been assumed to be absent [1]. It is only until recently

resonant excitonic effects and strong electronic correlations were reported in $SrTi_{1-x}Nb_xO_3$ family [2]. These excitonic effects can interact with a graphene layer through hybridizations at the interface. Many novel physical phenomena such as superconductivity, 2D electron gas, ferromagnetism, etc are reported to take place at the interfaces of perovskite oxides. Hence, a comprehensive experimental and theoretical study to investigate how the coupling between charge, lattice and orbital dynamics would result in the intriguing phenomena would be invaluable. Based on these studies, it is interesting that stacking monolayer-MoS_2 on STO substrates is expected to produce intriguing optical and electronic phenomena. These are the key motivations to revisit the fundamental understanding of heterointerfaces, particularly, between monolayer-MoS_2 and STO.

Spectroscopic ellipsometry measurements and absorption coefficient. We use a J. A. Woollam Co., Inc spectroscopic ellipsometer with photon energy of 0.6–4.5eV to measure the ellipsometry parameters Ψ (the ratio between the amplitude of *p*-and *s*-polarized reflected light) and Δ (the phase difference between of *p*-and *s*-polarized reflected light) in a high vacuum chamber with a base pressure of 1×10^{-9}mbar. The substrate layers (bulk $SrTiO_3$ or Al_2O_3) are also measured under the same conditions. The absorption coefficient of MoS_2 monolayer was extracted from the parameters Ψ and Δ utilizing an air/MoS_2/STO (or Al_2O_3) multilayer model, where monolayer-MoS_2 consists of a homogeneously uniform medium[3, 4] and a composite heterointerface component.

The Ψ and Δ defined as [5]

$$\tan\Psi\exp(i\Delta) \equiv \frac{r_p}{r_s} \qquad (S1)$$

where $r_{p(s)}$ is the reflectivity of *p*-(*s*-) polarized light. Using the Fresnel equations, the quantities could be defined as

$$r_p^{ij} = \frac{n_j\cos\theta_i - n_i\cos\theta_j}{n_j\cos\theta_i + n_i\cos\theta_j} \qquad (S2)$$

and

$$r_s^{ij} = \frac{n_j\cos\theta_i - n_i\cos\theta_j}{n_j\cos\theta_i + n_i\cos\theta_j} \qquad (S3)$$

Here *n* and *θ* represent the refractive index and angle of incident, respectively. The *i* and *j* represent the two materials. The complex dielectric function $\varepsilon(\omega) = \varepsilon_1(\omega) + i\varepsilon_2(\omega)$ of media can be obtained using

$$\sqrt{\varepsilon(\omega)} = n(\omega) \qquad (S4)$$

where *ω* is the photon frequency.

The absorption coefficient of media is obtained as

$$\alpha = \frac{4\pi k}{\lambda} \qquad (S5)$$

Here, *k* is the extinction coefficient (the imaginary part of the complex refractive index *n*) and λ is the light wavelength.

The reflectivity of MoS_2 film on substrate can be expressed as [6],

$$r_{multi} = \frac{r_{amb,MoS_2} + r_{MoS_2,sub}\exp\left(i2\delta_{MoS_2}\right)}{1 + r_{amb,MoS_2}\cdot r_{MoS_2,sub}\exp\left(i2\delta_{MoS_2}\right)} \qquad (S6)$$

where

$$\delta_{MoS_2} = \frac{2\pi d_{MoS_2}}{\lambda}\sqrt{n_{MoS_2}^2 - n_{amb}^2\sin^2\theta} \qquad (S7)$$

where subscripts multi and amb represent the MoS_2 on STO (or Al_2O_3) substrate multilayer system and the ambient, respectively, while δ_{MoS2} is the change in light phase as it reflects off the MoS_2 film, and d_{MoS2} is the thickness of the MoS_2 film which is used 0.7nm for the average of thickness of monolayer-MoS_2 grown by CVD. For the refractive index of the substrate, the spectroscopic ellipsometry were measured separately. The ex-situ ellipsometric measurements at room temperature are performed at three incident angles, 65, 70, 75 degrees. The similarity in fitting results demonstrates that the optical properties of the monolayer MoS_2 is generally angle independent within this angular range. Whereas, the in-situ temperature dependent ellisometric measurements are performed at 70 degree incident angle, due to the fixed angle of the cryo-chamber. The absorption coefficient of MoS_2 monolayer is obtained through direct function inversion of Equation S4 and S5.

Additional Analysis of Measured STO Exciton

Additional analysis of the measured STO exciton at ~3.8eV was performed with and without monolayer-MoS_2 to verify that exciton D is formed on monolayer-MoS_2 as a result of interfacial interactions instead of mere overlapping of optical signals.

We fixed the optical parameters of monolayer-MoS_2 as the reference (monolayer-MoS_2 on Al_2O_3 substrate). Using the new ellipsometric model (air/fixed reference-monolayer-MoS_2/variable-STO three-layer model), we extracted the variable STO optical parameters (STO with MoS_2) and compare it with the optical parameters of bulk STO (STO without MoS_2). The differences in the optical dielectric function at room temperature and low

temperature, ε_2, are shown in Fig. S2(a,b) (the optical spectra are normalized to clearly show the difference). Figure S2(c) (main text Fig. 2(e)) shows that there is a Wannier-like exciton at ~3.8eV for the STO without MoS_2. Comparing the STO signals with and without the MoS_2 in Figure S2a, we can clearly see that there is a stronger spectral feature at ~3.8eV for STO with MoS_2 compared to that of STO without MoS_2. Fig. S2(b), at low temperature, shows two split features at ~3.6eV and ~3.8eV for STO with MoS_2 unlike STO without $MoS_{2.}$ The stronger features in the former system (D and D' correspondingly) indicates they arise neither due to the reference bulk STO nor the free-standing MoS_2. Instead, we can deduce that the additional features are attributed to interfacial interactions between MoS_2 and STO.

We conclude that the change in exciton at ~3.8eV is not a mere overlap of optical signals from monolayer-MoS_2 and STO substrate. Instead, it is an optical signature which indicates that the exciton arises due to the interaction at the heterointerface.

A further evidence that the exciton is not a mere overlap of optical signals is that the new exciton in the optical data of the MoS_2/STO sample resulted in the onset of two optical peaks (at ~3.6 and ~3.8eV) at 77K, which can be attributed to the spin-orbit coupling in MoS_2. In contrast, there is only one optical peak located at ~3.8eV for bulk STO. This further supports our claim that there is propagation of excitonic effects through the interface.

Estimation of energy positions of features from valence band and work function spectra: The broadening of the valence band and work function photoemission spectra can be caused by some sample features, namely, surface roughness, sample defects, impurities and vacancies. In situations when the peak is broad, defining the energy positions of features to be located at the middle of the edge would undermine its accuracy. Hence, the estimate energy positions of apparent valence band maximum (VBM*) and work function in Figures 4(b) and 4(c) are derived by intersecting the extrapolated leading edge with the background baseline. Prior to measurement, the photon energy was calibrated using Fermi-edge position of a sputter-cleaned gold foil in electrical contact with the sample. This calibration is also derived by intersecting the extrapolated leading edge with the background baseline (0 eV), instead of the middle of the Fermi-edge.

The effect of the different intact between MoS_2 layer and STO: From the experimental perspective and to consider the quality of our samples, with the large beam spot size (order of 1 millimeter) that of Spectroscopic Ellipsometry, the XAS and the XPS, these experimental techniques register an averaged signal from the sample. Hence, as long as some part where the MoS_2 and substrate is intact with the interfacial hybridization, the onset of the exciton and the interfacial effects can be detected.

GW-BSE Calculations of MoS_2 on STO with Different configurations

To further substantiate that there is agreement between experiment and theory, we performed first-principle studies involving monolayer-MoS_2 in two different interfacial atomic positions (both the MoS_2 monolayers being shifted from the most stable position), as shown in Figure S7. This provides further verification that interfacial hybridization and formation of the new exciton still takes place even with the stacking of MoS_2 and STO in different configurations as long as there are strong interfacial interactions between the MoS_2 and STO substrate.

The most stable configuration for MoS_2 monolayer on STO (001) substrate is the interfacial structure with a maximized potential bonding. The most stable structure is shown in Figure S8(a), where most S atoms are located on the top of the Ti atoms, and the Mo atoms reside on the top O atoms at the STO surface. This stable monolayer-MoS_2/STO structure gives us an additional excitonic peak at ~4 eV (see Figure S8(b)), this is once again due to the interfacial orbital hybridization as discussed in the main text. A metal-stable interfacial configuration is shown in Figure S8(c), which has similar interfacial bonding configuration, but its adsorption energy is slightly higher (~60 meV) than that of the structure in Figure S8(a). Interestingly, with the GW-BSE calculation, this metal-stable structure also shows the high-energy excitonic peak (see Figure S8(d)). This shows that the onset of the new exciton is attributed to the unique interfacial hybridization which is weakly related to how the TMDs placed on the STO.

High-Energy Excitons D and D' in high-energy photoluminescence spectra. For steady-state photoluminescence measurement, a liquid-nitrogen cooled Linkam stage (FTIR600) is used to cool the sample temperature to 77K and the emission spectra are

measured with a Fluorolog-3, (HORIBA Jobin Yvon) coupled with Synapse CCD camera. The photoluminescence excitation source for both the STO and MoS2/STO samples is constant at 295nm (~4.2eV) and the PL signal was collected from the front by focusing the signals into a fiber coupled spectrometer (Ocean Optics Maya pro 2000). All high-energy PL temperature-dependent measurements were carried out in vacuum.

To extract the profiles of the excitonic signals of monolayer-MoS_2 from that of the thick STO substrate at each corresponding temperature, each set of PL data is normalized to the respective maximum of the MoS_2/STO spectrum multiplied by a constant value to match the maximum intensity of the STO reference spectrum. The normalized spectrum is then extracted (by subtracting spectra with and without MoS_2) to visualize the excitonic peaks (Figures S5 and S6).

Note that as compared to the bulk STO reference PL spectrum, the signal-to-noise ratio of the MoS_2/STO PL spectrum is relatively lower due to variation in the alignment of the MoS_2/STO sample. This results in a seemingly stronger PL excitation source peak as compared to that of the STO substrate which requires an additional fitting peak to be accounted for. The PL spectral difference is then modelled using two Gaussian lineshapes – for both exciton peaks D and D'. The excitonic peaks at their respective temperatures are provided in Figure S4. They are consistently present in all investigated temperatures, suggesting that these are not experimental artefacts but real signals that arise. A further indication of the presence of these novel many-body phenomena as detected by spectroscopic ellipsometry.

Supplementary Figure S6(a) compares the normalized PL data between MoS_2/STO and STO at 77K. The maximum of the MoS_2/STO spectrum is normalized with the maximum intensity of the STO reference spectrum. Although both spectra are similar with a prominent excitonic signal that the STO substrate displays, marked differences are observed at the ~3.60eV (see figure inset) and ~3.80eV region. Besides, these spectral differences are consistently present with repeated measurements at different temperatures (Figure S5). The spectral differences between monolayer-MoS_2/STO and STO are studied by a fitting analysis using two Gaussian lineshapes as displayed in Figure S6(b). Interestingly, the peak positions and widths of the lineshapes are consistent with the profiles of peaks D and D' elucidated from spectroscopic ellipsometry data. Analytical results are strong indications pointing to the existence of high-energy excitons. Despite monolayer-MoS_2 (~1nm) being much thinner than the STO substrate, the PL signal suggests an influential effect of the high-energy interfacial excitonic responses on the radiative properties of monolayer-MoS_2. Note that the PL excitation source for both the STO and MoS_2/STO samples is constant at 295nm (~4.2eV). As compared to the bulk STO reference PL spectrum, the signal-to-noise ratio of the MoS_2/STO PL spectrum is relatively lower due to variation in the alignment of the MoS2/STO sample. This results in a seemingly stronger PL excitation source peak as compared to that of the STO substrate which requires an additional fitting peak to be accounted for.

High-Energy Excitons D and D' in spectroscopic ellipsometry. To elucidate additional further insights on how the excitonic transitions evolve with temperature, the section of the absorption spectra within energy range ~3.3-4.2 eV has been extracted and modelled

using mixed Lorentzian-Gaussian lineshapes. Two oscillators D and D' each with a combination of Lorentzian (30%) and Gaussian (70%) components have been used to model the absorption spectra at temperatures between 77 and 350K.

Based on the temperature-dependence of the excitonic peak profiles, the binding energies of excitons D and D' can be elucidated. The absorption linewidth, Δv, of each excitonic peaks can be described as the sum of its temperature-independent and temperature-dependent components as shown[7]

$$\Delta v = \Delta v_0 + v_T \exp\left(\frac{E_B}{kT}\right) \qquad (S8)$$

Where Δv_0 represents the temperature-independent linewidth; v_T denotes the attempted frequency for excitonic thermal dissociation, and E_B is the exciton binding energy. The equation has been rearranged into the following form for the purpose of linear fitting:

$$\ln(h\Delta v - h\Delta v_0) = \ln hv_T - \frac{E_B}{kT}$$

$$\ln(\Delta E - \Delta E_0) = \ln E_T - \frac{E_B}{kT} \qquad (S9)$$

Where $\ln(\Delta E\text{-}\Delta E_0)$ is plotted as a function of 1/T. ΔE_0 is assumed to be ~78.11meV for exciton D and ~56.67meV D' based on the low-temperature linewidth. The linewidth at each temperature can be calculated based on the first-order derivative of the absorption peak and measuring between the peak and zero-crossing and then doubled to obtained the its full-width at half-maximum (FWHM) for each corresponding temperature[7].

However, due to the overlapping of multiple features within the energy range and that the excitonic peaks become less distinct with rising temperature, it is not possible to elucidate

the linewidth from the data using this technique. Hence, the temperature-dependent excitonic linewidths are instead estimated using the FWHM profiles of the Lorentzian-Gaussian lineshapes.

The natural logarithm of the excitonic transition linewidths of MoS_2/STO are plotted in Figure S3 as functions of the inverse temperature. By fitting the data with Equation S9, it yields a binding energy of (13.66±1.64)meV for exciton D and (14.48±1.37)meV for exciton D' – about an order of magnitude smaller than the low-energy excitons[8].

The respective estimated binding energies are further verified by considering the temperature dependence of the spectral weights of exciton D and D' (Figure S4). Note that there is a drastic increase in spectral weight in the ~ 200 K (~0.17meV) temperature regions for both the excitons. While there are minor spectral weight increases in the temperature range above and below, these drastic rises in spectral weight suggest the dissociation of excitons into their individual charged constituents above their respective binding energies. This results in the significant increase in charge carrier population above this temperature range.

Supplementary References

[1] M. Capizzi, A. Frova, *Phys. Rev. Lett.* **1970**, *25*, 1298.

[2] P. K. Gogoi, L. Sponza, D. Schmidt, T. C. Asmara, C. Diao, J. C. W. Lim, S. M. Poh, S.-i. Kimura, P. E. Trevisanutto, V. Olevano, A. Rusydi, *Phys. Rev. B* **2015**, *92*, 035119.

[3] X. Yin, M. A. Majidi, X. Chi, P. Ren, L. You, N. Palina, X. Yu, C. Diao, D. Schmidt, B. Wang, P. Yang, M. B. H. Breese, J. Wang, A. Rusydi, *NPG Asia Mater.* **2015**, *7*, e196.
[4] X. Yin, S. Zeng, T. Das, G. Baskaran, T. C. Asmara, I. Santoso, X. Yu, C. Diao, P. Yang, M. B. H. Breese, T. Venkatesan, H. Lin, Ariando, A. Rusydi, *Phys. Rev. Lett.* **2016**, *116*, 197002.
[5] H. Fujiwara, *Spectroscopic ellipsometry: principles and applications*, John Wiley & Sons, **2007**.
[6] B. Harbecke, *Appl. Phys. B* **1986**, *39*, 165.
[7] V. D'Innocenzo, G. Grancini, M. J. P. Alcocer, A. R. S. Kandada, S. D. Stranks, M. M. Lee, G. Lanzani, H. J. Snaith, A. Petrozza, *Nat. Commun.* **2014**, *5*, 3586.
[8] H. M. Hill, A. F. Rigosi, C. Roquelet, A. Chernikov, T. C. Berkelbach, D. R. Reichman, M. S. Hybertsen, L. E. Brus, T. F. Heinz, *Nano Lett.* **2015**, *15*, 2992.
